# Autophagy in Macrophages: Impacting Inflammation and Bacterial Infection

**DOI:** 10.1155/2014/825463

**Published:** 2014-04-09

**Authors:** Ali Vural, John H. Kehrl

**Affiliations:** B-Cell Molecular Immunology Section, Laboratory of Immunoregulation, National Institute of Allergy and Infectious Diseases, National Institutes of Health, Building 10, Room 11N214, Center Drive, MSC 1876, Bethesda, MD 20892, USA

## Abstract

Macrophages are on the front line of host defense. They possess an array of germline-encoded pattern recognition receptors/sensors (PRRs) that recognize pathogen-associated molecular patterns (PAMPs) and which activate downstream effectors/pathways to help mediate innate immune responses and host defense. Innate immune responses include the rapid induction of transcriptional networks that trigger the production of cytokines, chemokines, and cytotoxic molecules; the mobilization of cells including neutrophils and other leukocytes; the engulfment of pathogens by phagocytosis and their delivery to lysosome for degradation; and the induction of autophagy. Autophagy is a catabolic process that normally maintains cellular homeostasis in a lysosome-dependent manner, but it also functions as a cytoprotective response that intersects with a variety of general stress-response pathways. This review focuses on the intimately linked molecular mechanisms that help govern the autophagic pathway and macrophage innate immune responses.

## 1. Introduction

Ubiquitin-proteasome system and lysosomes are the intracellular degradation units of eukaryotic cells. Macroautophagy (hereafter referred as autophagy) is defined as a catabolic process maintaining cellular homeostasis in a lysosome-dependent manner [1]. The process of autophagy includes sequestration of long-lived proteins and bulky cytosolic contents into double-bilayer vesicular compartments followed by their delivery to lysosomes for degradation [2]. The final metabolites of lysosomal activity are then reused to fulfill energy and new macromolecule needs of the cell. The autophagic process functions as an intracellular recycling mechanism [3]. Autophagic machinery is activated in response to various cellular stresses and often has a cytoprotective function [4]. Depending on the nature of the trigger, either autophagy may proceed as a nonselective bulk degradation process or selectively labeled substrates may be targeted for degradation [5]. Nutrient deprivation, damaged or excessive organelles, accumulated misfolded proteins, endoplasmic reticulum stress, oxidative stress, certain toxins, radiation, and hypoxia can all trigger autophagy [4]. The reactive nature of autophagy gives rise to its participation in a wide array of physiologic and pathologic pathways involved in cell survival, tumor suppression, lifespan extension, cell death, cell differentiation, organismal development, and immunity [6, 7]. As a consequence defects in autophagic machinery can cause or contribute to cancer, neurodegenerative diseases, myopathies, immune deficiencies, and premature aging [6].

**Figure 1:**
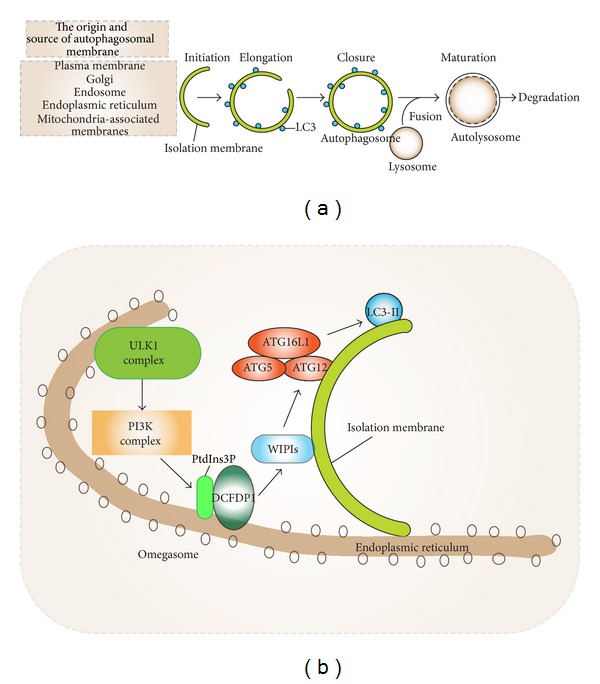
(a) The general scheme of autophagic process is shown. Autophagy is defined as the sequestration of substrates into double-bilayer membrane vesicles termed autophagosomes for degradation. The autophagic process starts with the formation of isolation membrane (phagophore) that originates from various intracellular membrane sources. Initiation of the isolation membrane is followed by elongation and closure leading to a complete autophagosome that surrounds the cargo. The fusion of lysosomes with autophagosomes causes the formation of autolysosomes, where autophagic substrates are exposed to hydrolytic interior of lysosome resulting in their degradation. (b) The molecular representation of autophagy initiation is shown at phosphatidylinositol-3-phosphate- (PtdIns_3_P-) positive membrane structures named “omegasomes.” The induction of autophagy translocates ULK1 complex to the endoplasmic reticulum leading to activation of the PtdIns_3_P kinase (VPS34/Beclin-1/ATG14L) complex. VPS34-derived PtdIns_3_P recruits double FYVE-containing protein 1 (DFCP1/ZFYVE1) and WD-repeat protein interacting with phosphoinositides (WIPIs) to the outer membrane of autophagosomes causes the association of the ATG5/ATG12 conjugate with ATG16L1. The ATG5/ATG12/ATG16L1 complex then adds phosphatidylethanolamine group to the C-terminus of the LC3 protein promoting the elongation of isolation membrane.

The hallmark of autophagy is the formation of double-membrane vesicles called autophagosomes. The autophagic process consists of four main steps: (1) initiation, (2) elongation of autophagosomes, (3) closure, and (4) fusion with lysosomes [8]. The sources of autophagosome membrane and the factors underlying autophagosome membrane dynamics are complex and a substantial body of literature has addressed their initial formation [3, 9–11]. Autophagosomes emerge in the cytoplasm as an autophagic phagophore (isolation membrane) at cup shaped protrusions termed omegasomes. These often arise from the endoplasmic reticulum (ER) at sites rich in phosphatidylinositol-3-phosphate (PtdIns_3_P) and double FYVE-containing protein 1 (DFCP1). The alternative name of DFCP1 is zinc finger FYVE domain-containing protein 1 (ZFYVE1) [9]. The Golgi apparatus, mitochondria-ER contact sites, and plasma membrane derived endocytic organelles also support phagophore formation. A large group of proteins assist in autophagosomal biogenesis. These proteins were initially characterized in yeast and designated autophagy-related genes (ATGs) proteins [1]. See Figure 1 for a brief outline of the different stages in autophagosome formation.

Several key molecular events have emerged from the study of starvation induced autophagy. The mTOR complex 1 (mTORC1) regulator is a major sensor of the energy and nutrient status of the cell [12]. Upon activation, mTORC1 phosphorylates ATG13 preventing it from entering the UNC-51 like serine/threonine kinase complex (ULK1 kinase complex). This blocks autophagy. Inhibition of mTORC1 leads to the activation of the ULK1 kinase complex. This links upstream signals to the core autophagy machinery as Beclin-1 is a ULK1 substrate. The PtdIns_3_P kinase VPS34/Beclin-1/ATG14L complex can then funnel signals to two downstream conjugation systems: ATG5/ATG12/ATG16L1 and ATG7/ATG3/ATG8-LC3 (microtubule-associated light chain 3, GABARAP) [13]. The former adds a phosphatidylethanolamine group to the carboxyl terminus of ATG8 paralogs. This results in lipid conjugation of LC3 into phagophore membrane as LC3-II and is useful as a mammalian autophagic marker. Consequently, ATG8 along with additional factors promotes the elongation and closure of the phagophore, thereby forming the double membrane autophagosome. After that, the autophagosomes can fuse with lysosomes, gaining the capacity to digest their contents by the acquisition of lysosomal hydrolytic enzymes [14]. The fusion is mediated by the translocation of the SNARE protein syntaxin 17 to the outer membrane of autophagosomes [15]. We refer the reader to other comprehensive reviews covering the complex and dynamic initiation mechanisms of autophagy [1, 6, 9–11].

## 2. Macrophage Pattern Recognition Receptors (PRRs): Gatekeepers of Autophagy Activation during Innate Immune Responses

The autophagic response provides cytoprotective and homeostatic functions and intersects with a variety of general stress-response pathways, and recent studies have revealed an intimate linkage between the autophagic pathway and various innate immune responses. These include assisting in the elimination of invading pathogens, impacting pathogen recognition via PRRs, regulating inflammasome-dependent signals, and affecting phagocytosis [16]. Defects in autophagic machinery can worsen or directly contribute to various infectious diseases and inflammatory syndromes [17]. Given such a substantial contribution to innate immunological processes by autophagy, it has been described as an emerging immunological paradigm [18].

Macrophages constitute a critical cell type in the innate immune response [19, 20]. They are equipped with germline-encoded pattern recognition receptors/sensors (PRRs) that aid in the recognition of various moieties from microbes termed pathogen-associated molecular patterns (PAMPs) and also danger-associated molecular patterns (DAMPs) [21]. Lipids, nucleic acids, proteins, lipoproteins, glycans derived from a range of bacteria, viruses, parasites, and fungi are designated as PAMPs. Depending on the specific receptor-PAMP/DAMP match and whether multiple PRRs are engaged, various downstream effectors/pathways are activated, which prepare the cell to combat the invading agents by activating degradation pathways and relaying signals such as cytokines to alert other cells of the innate and adaptive immune system in the surrounding tissues and at distal sites [4, 22, 23].

### 2.1. Toll-Like Receptors (TLRs)

The discovery of* Drosophila* Toll as a PRR in antifungal defense led to identification of TLR homologues in mammalians [24–26]. TLRs, which constitute one subgroup of PRRs, are a type I transmembrane protein. Structurally TLRs are composed of extracellular portion, which contains leucine-rich repeats responsible for the recognition of PAMPs; the transmembrane domain; and the intracellular Toll/interleukin-1 (IL-1) receptor (TIR) domains, which mediate downstream signaling [27]. To date, 13 TLRs have been identified in mice and 10 in humans. TLRs are positioned either at the cell surface or on the lumen of intracellular vesicles. TLR1, TLR2, TLR4, TLR5, TLR6, and TLR10 are localized on the plasma membrane and recognize lipids, lipoproteins, and proteins. TLR3, TLR7, TLR8, and TLR9 are localized in intracellular vesicles such as the endoplasmic reticulum (ER), endosomes, lysosomes, and endolysosomes and they detect microbial nucleic acids [27].

TLR2 recognizes lipopeptides, peptidoglycan, lipoteichoic acid, and zymosan derived from pathogens. In addition, TLR2 forms heterodimers with TLR1 and TLR6. Such dimerization provides specificity for the detection of certain lipoproteins. TLR4 detects lipopolysaccharide (LPS), a major bacterial signature molecule found on the outer membrane of Gram-negative bacteria. TLR5 recognizes the flagellin protein, a major component of bacterial flagella. TLR3 detects double-stranded RNA (dsRNA) of RNA viruses and a synthetic analog polyinosinic-polycytidylic acid (poly(I:C)). TLR7 and human TLR8 recognize single-stranded RNA of RNA viruses and imidazoquinoline derivatives such as imiquimod and resiquimod (R-848) and guanine analogs. TLR9 recognizes unmethylated 2′-deoxyribo(cytidine-phosphate-guanosine) (CpGs) DNA motifs that are frequently present in viral DNA. TLR10 ligand is still unknown.

The binding of PAMPs to TLRs initiates innate immune response and helps prime antigen-specific adaptive immunity. Activation of different TLRs stimulates signal transduction pathways that lead to distinctive biological responses as different adapter proteins are recruited to distinct TLRs. This leads to the activation of downstream effectors that determine the diversity of the response. The known TLR adapter proteins are myeloid differentiation factor 88 (MyD88); TIR domain-containing adapter-inducing interferon-*β* (TRIF); MyD88 adapter-like (Mal), also termed TIRAP; TRIF-related adaptor molecule (TRAM); and sterile *α*- and armadillo motif-containing protein (SARM) [28]. MyD88 is recruited by all TLRs except TLR3 and activates the transcription factor nuclear factor-*κ*B (NF-*κ*B) and mitogen-activated protein kinases (MAPKs), whose major functions are to induce inflammatory cytokines. TRIF is recruited by TLR3 and TLR4 and activates interferon regulatory factor-3 (IRF3) and NF-*κ*B with the consequent induction of type I interferon and inflammatory cytokines [27].

### 2.2. The MyD88-Dependent Pathway

MyD88 is among the best studied of the TLR adapters. It is a death domain- (DD-) containing cytosolic protein, which is recruited to activated TLRs and adopts a hexameric form that leads to the further recruitment of death domain- (DD-) containing kinases including IL-1 receptor- (IL-1R-) associated kinase 1 (IRAK1) and IRAK4 [28]. Activation of IRAKs through phosphorylation increases the association with an E3 ubiquitin ligase and scaffolding protein and tumor necrosis factor receptor- (TNFR-) associated factor 6 (TRAF6). TRAF6 catalyzes K63-linked polyubiquitination of IRAK1 and of itself. TRAF6 then binds through these ubiquitin proteins to transforming growth factor-*β*- (TGF-*β*-) activated protein kinase 1 (TAK1) and TAK1-binding protein (TAB1) and leads to phosphorylation of the inhibitor of nuclear factor- (NF-) *κ*B (I*κ*B) kinase (IKK) complex. As a result, I*κ*B is degraded freeing NF-*κ*B to translocate to the nucleus to induce transcription of inflammatory cytokine genes. In addition it induces A20 expression, which negatively regulates the activation of NF-*κ*B in part by deubiquitinating TRAF6 [29, 30].

### 2.3. Initial Evidence That Bacterial Infection Triggers Autophagy

A decade ago several studies revealed a link between autophagy activation and bacterial infection. Nakagawa et al. demonstrated the induction of autophagy in nonphagocytic cells (HeLa cells) following infection with* Streptococcus pyogenes* (Group A* Streptococcus*, GAS) acted as a defense mechanism [31]. The bacteria were found to colocalize with LC3 and LAMP-1 positive vesicles and markers of autophagosomes and lysosomes, respectively. Moreover, autophagy deficient (ATG5^−/−^) cells infected with GAS yielded higher rates of bacterial viability suggesting that autophagy helps eliminate the bacteria following fusion of autophagosomes with lysosomes [31]. Later, a similar phenomenon was observed in* Mycobacterium tuberculosis *infected macrophages [32].* M. tuberculosis *inhibits the maturation of phagosomes by interfering with the phagosome maturation pathway. The induction of autophagy led to colocalization of LC3 and Beclin-1 with* M. tuberculosis *containing phagosomes indicating their maturation into phagolysosomes. Moreover,* M. tuberculosis *survival rates were reduced following autophagy induction in infected macrophages suggesting that the degradation of* M. tuberculosis* containing phagosomes in a lysosome-dependent manner overcame the trafficking block imposed by* M. tuberculosis *[32].

### 2.4. TLR-Induced Autophagy

Based on the studies showing the induction of autophagy following bacterial infection and the initial evidence reporting the link between TLR4 and autophagy [33], our group hypothesized that the engagement of TLRs by bacterial products might provide an inductive signal for autophagosome formation in macrophages. To test this idea, we engineered a macrophage cell line RAW264.7 to stably express green fluorescent protein (GFP) linked to LC3 (GFP-LC3). Upon starvation green dots corresponding to induced autophagosomes could be visualized and measured. Next, we treated this cell line with different PAMP ligands that engaged the known TLRs and measured autophagosome formation [34]. With the exception of TLR9, engagement of the other TLRs induced autophagy in these cells. The adapter molecules that transduced the TLR3/4 dependent signals were determined as MyD88 and TRIF. TLR4 immunoprecipitation using a TLR4 agonistic antibody led to the coimmunoprecipitation of Beclin-1, TRIF, IRAK4, and MyD88. The death domain of MyD88 proved essential for Beclin-1 recruitment. In addition, triggering TLR3, TLR4, and TLR7 led to a dissociation of Beclin-1 from its antiapoptotic and antiautophagy binding partner Bcl-2 [34].

The induction of autophagy through PAMP-activated TLR signaling was also demonstrated by two other groups with a few different nuances [33, 35]. Xu et al. found receptor-interacting protein (RIP1) and p38 mitogen-activated protein kinase as the downstream effectors of LPS-induced TLR4-dependent autophagic pathway. The adapter TRIF was shown to transduce the signal but not MyD88. LPS-induced autophagy proceeded through the association of VPS34, a Class III PI3K with membranes [33]. Delgado et al. extended the scope of TLR-induced autophagy examining a range of TLR ligands and demonstrating the activation of autophagy in murine primary bone marrow-derived macrophages (BMDM), RAW264.7, and J774 cells. The focal point of the study was the induction of autophagy through TLR7 via single-stranded RNA and imiquimod ligands [35]. Beclin-1 was shown to be critical for TLR7-dependent autophagic activation, and MyD88 was shown as a downstream adapter of TLR7-dependent signaling. The knockdown of each protein (i.e., TLR7, MyD88, and Beclin-1) impaired the clearance of the intracellular microbe* M. tuberculosis *var*. bovis *Bacille Calmette-Guerin (BCG). Furthermore treatment with imiquimod and ssRNA enhanced the degradation of the pathogen via TLR-mediated autophagic activation [35].

**Figure 2:**
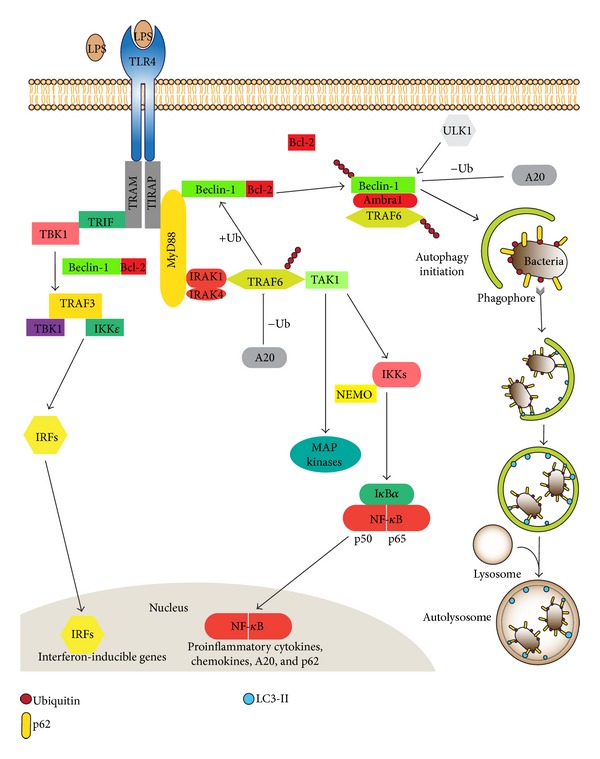
The downstream molecular pathways following the activation of TLR4 receptor by lipopolysaccharide (LPS) are shown. The adapter protein MyD88 is recruited by TLR4 and activates the transcription factor nuclear factor-*κ*B (NF-*κ*B) and mitogen-activated protein kinases (MAPKs), whose major functions include the induction of proinflammatory cytokines, chemokines, A20, and p62. TRIF is another adapter protein recruited by TLR4. It causes the activation of interferon regulatory factor-3 (IRF3) and NF-*κ*B leading to induction of type I interferon and inflammatory cytokines. In addition, LPS-induced TLR4 activation recruits Beclin-1 through adapter proteins MyD88 and TRIF leading to formation of autophagosomes. The ubiquitination status of Beclin-1 is regulated by the TRAF6/A20 axis, which has a regulatory role in the induction of autophagosomes in response to pathogens. Pathogens can be ubiquitinated and thereby recruit autophagic adaptors like p62.

Further study of the control mechanisms that regulate TLR-induced autophagy led to the finding that Beclin-1 underwent K63-linked ubiquitination [29, 30]. As indicated previously K63-linked ubiquitination is involved in numerous cells signaling pathways, in stress responses, and in the intracellular trafficking of membrane proteins [36]. TRAF6 bound Beclin-1 and mediated K63-linked ubiquitination following TLR4 stimulation. On the contrary, A20, a deubiquitinating protein of TRAF6, decreased Beclin-1 ubiquitination. Furthermore, a key lysine residue (K117) in Beclin-1 served as a site of K63-linked ubiquitination. Moreover, the ubiquitination at this site promoted the oligomerization of Beclin-1 and influenced the autophagic state in a PI3K activity-dependent manner. The functional significance of K63-linked Beclin-1 ubiquitination was later elucidated using the stable GFP-LC3 expressing RAW264.7 cells. TRAF6 mRNA silencing decreased the number of autophagic vesicles, whereas A20 knockdown increased them. In addition to LPS-induced TLR-mediated autophagy, Beclin-1 ubiquitination was also triggered following treatment with IL-1 or IFN-*γ* and following amino acid starvation, all of which lead to induction of autophagy. These data suggested that the ubiquitination of Beclin-1 likely functions to trigger the formation of autophagosomes in response to a number of different stimuli [37]. See Figure 2 for a schematic of TLR signaling induced autophagosome formation.

In addition to certain overlapping findings with other groups, our studies captured the recruitment of Beclin-1 to adapter proteins MyD88 and TRIF following TLR activation [34]. The interaction of Beclin-1 is reduced with antiapoptotic Bcl-2 protein following TLR activation suggesting a possible crosstalk between autophagy and apoptosis pathways [34]. The mobility shift of Beclin-1 protein band following TLR activation led to the discovery that Beclin undergoes TRAF6 mediated K63-linked ubiquitination and a major ubiquitination site in Beclin-1 (K117) was identified. A20 functioned to deubiquitinate TRAF6 and Beclin-1. The K63 ubiquitination of Beclin-1 may serve to multimerize Beclin-1 enhancing the lipid kinase activity of PI3KC3 and augmenting TLR-induced autophagy in macrophages, while A20 negatively regulates TRAF6 and Beclin-1 opposing TLR-induced autophagy [29, 30].

Macrophages are challenged with LPS form transient cytosolic aggregation of ubiquitin-positive bodies called aggresome-like induced structures (ALIS) [38, 39]. Fujita et al. investigated the molecular dynamics of ALIS formation and its relationship to autophagy in macrophages. As LPS induced autophagosome-like structures even following ATG5 and ATG7 knockdowns, their induction appeared not to depend upon the classical autophagic pathway. The adapter protein sequestosome 1 (p62/SQSTM1) recruited both LC3 and ubiquitin to ALIS. p62 links ubiquitinated substrates to autophagosomes by virtue of binding both ubiquitin and LC3 (see discussion of xenophagy, Section 3). The knockdown of p62 led to a loss of LC3 and ubiquitin body formation, and ALIS increased. Furthermore, the knockdown of MyD88, TRAF6, TRIF, and IRAK4 all decreased LPS-induced autophagosome formation and downregulated the p62 mRNA suggesting that MyD88-dependent TLR4 signaling was essential for p62 induction and ALIS formation. Nrf2 (nuclear factor erythroid 2-related factor 2), a downstream effector of ROS-p38 axis, was found to upregulate p62 expression [40, 41]. TLR4 signaling upregulated Nrf2, which increased p62, leading to the assembly of ALIS, and the subsequent autophagic degradation of ALIS [41]. Moreover, it revealed a potential convergence of the innate immune response and autophagy via oxidative stress [40]. Subsequently, it was also shown that ALIS formation strictly depended upon p62, NF-*κ*b, and mTOR proteins. However, this study suggested that ALIS clearance did not depend on canonical nor noncanonical autophagy pathways but did depend upon lysosomes [42, 43].

### 2.5. NOD-Like Receptors (NLRs) and Inflammasomes

NLR pathways are prominently involved in recognizing danger signals of endogenous and exogenous origins [44]. The NLR family consists of 22 cytoplasmic proteins corresponding to the 5-member NOD (nucleotide-binding oligomerization domain) family, 14 NLRPs, IPAF, NAIP, and CIITA [45, 46]. NOD proteins recognize bacterial cell wall components (i.e., peptidoglycans) in the eukaryotic cell's cytosol. Activation of NOD1 and NOD2 by muramyl dipeptides, a peptidoglycan constituent of both Gram-positive and Gram-negative bacteria, activates autophagy by recruiting ATG16-like 1 (ATG16L1) to the plasma membrane at the bacteria entry site. This leads to efficient bacterial sequestration in autophagosomes and subsequent bacteria degradation [47]. Polymorphisms in* ATG16L1* and* NOD2* genes have been linked to Crohn's disease, an intestinal inflammatory disease. Cells obtained from Crohn's disease patients with the* ATG16L1 (T300A)* polymorphism have decreased autophagic activity following exposure to muramyl dipeptides. In addition, a truncated version of NOD2 found in some patients with Crohn's disease cells leads to the retention of ATG16L1 in cytoplasm, inhibiting its recruitment to plasma membrane and reducing autophagic activity [48].

Inflammasomes are multimeric protein complexes that activate caspase-1. They are assembled following the detection of a variety of cytosolic threats including infection, tissue damage, and metabolic abnormalities [49–51]. They consist of a sensor molecule (a NLR protein), an adaptor molecule ASC, and caspase-1 [52]. Most NLR proteins have an amino-terminal caspase-recruitment-and-activation domain (CARD) or a pyrin domain; a Nod (or NACHT domain) that mediates self-oligomerization; and carboxyterminal leucine-rich repeats (LRRs), which sense specific stimuli. Following their activation, NLRs oligomerize via their NACHT domains and connect to caspase-1 via the adaptor protein ASC, which consists of a pyrin domain and a CARD domain [53]. ASC interacts with the upstream NLR sensor molecules via its pyrin domain. This interaction leads to the assembly of ASC dimers and oligomers that can sometimes be visualized as a large cytosolic speck [54]. The CARD domain of ASC recruits procaspase-1 monomers, which leads to the cleavage of the proform and the assembly of the active heterotetrameric caspase-1 [55]. Once activated, caspase-1 cleaves the proinflammatory cytokine precursors prointerleukin-1*β* (pro-IL-1*β*) and pro-IL-18. This causes the production of the biologically active forms of IL-1*β* and IL-18, which are released from the cell by an unconventional secretory pathway [52].

### 2.6. Autophagy and Inflammasomes

The association between Crohn's disease and ATG16L1 polymorphisms ignited further investigations regarding the regulation of the inflammatory response by autophagic machinery [47]. To assess such a potential implication, Saitoh et al. generated an ATG16L1-deficient mouse strain. This results in a failure to recruit the ATG12-ATG5 conjugate to isolation membranes and impairs the conjugation of LC3-I to phosphatidylethanolamine, leading to total absence of autophagosomes and a significant reduction in autophagy-dependent degradation [56]. To assess the consequences of defective autophagy, macrophages from wild type and ATG16L1-deficient mice were treated with LPS for 24 hours. Although TNF*α*, IL-6, and IFN-*β* production were unchanged, the level of IL-1*β* was markedly elevated. Furthermore, higher IL-1*β* levels were observed following the exposure of ATG16L1-deficient macrophages to ATP or to monosodium urate (MSU), known as NLRP3 inflammasome activators. Besides IL-1*β*, elevations in IL-18 and active caspase-1 levels were observed in the ATG16L1 deficient macrophages. Similar results were found with ATG7-deficient macrophages. These studies indicate that impaired classical autophagy in macrophages elevates the production of inflammasome-specific cytokines, which suggested a regulatory action for the autophagic machinery on inflammasome activity [56].

Further studies focused on how autophagy regulated IL-1*β* secretion. Harris et al. found that pro-IL-1*β* is targeted by autophagosomes and degraded following exposure of macrophages to various TLR agonists [57]. Another study investigated inflammasome activity in macrophages from mice deficient in other autophagy-related proteins. Primary macrophages from mice lacking LC3 or from mice lacking one normal Beclin-1 allele secreted more IL-1*β* and IL-18 than did those prepared from wild type mice [58]. The deficiency of autophagy-related LC3 and Beclin-1 proteins deleteriously affected mitochondrial homeostasis resulting in increased basal ROS production and enhanced the release of mitochondrial DNA (mtDNA) into the cytosol following NLRP3 activation. Moreover, suggesting an* in vivo* consequence of this inflammasome dysregulation, these mice were more susceptible to bacterial sepsis following cecal ligation and puncture [58].

Our group elucidated a direct linkage between inflammasome activity and autophagy [59]. Using a THP-1 human monocytic leukemia cell line stably expressing GFP-LC3, we showed that the activation of AIM2 and NLRP3 inflammasomes led to the formation of autophagosomes in a Beclin-1-dependent manner. The inflammasome component ASC and AIM2 or NLRP3 sensor proteins exhibited partial colocalization with autophagosomes and autophagolysosomes. The manipulation of autophagy by activators (starvation, rapamycin) and inhibitors (3-methyladenine) during AIM2 or NLRP3 inflammasome activation altered the functional outcome of inflammasomes (i.e., the amount of the cleaved forms of IL-1*β* and caspase-1) [59]. Activation of autophagy shifted inflammasome components to an autophagic cytosolic fraction lowering mature IL-1*β* and caspase-1, whereas inhibition of autophagy led to accumulation of inflammasomes and elevated IL-1*β* and active caspase-1. These data suggested that the autophagic pathway acted to limit inflammasome activity by engulfing and degrading them. To understand how inflammasomes were selected and targeted to autophagosomes, we tested the role of the adaptor protein p62. We found that the knockdown of p62 in inflammasome-induced macrophages resulted in increased amounts of mature IL-1*β* and caspase-1. Moreover, p62 colocalized with ASC and immunoprecipitated with ASC and Beclin-1 following inflammasome induction. The inflammasome adaptor protein ASC was ubiquitinated and inflammasome complexes were earmarked as autophagic substrates by p62 upon inflammasome induction [59, 60]. Finally a mechanism linking inflammasome activation to the induction of autophagy was found. The small GTPase RalB and its effector Exo84 are known to be required for starvation-induced autophagy and RalB activation is sufficient to promote autophagosome formation [60, 61]. We found that RalB was activated upon exposure of cells to inflammasome activators, thereby providing a link between inflammasome activation and the induction of autophagy [59]. In addition, reducing RalB activation enhanced inflammasome activity increasing IL-1*β* secretion. The relationships between autophagy and inflammasome have been recently discussed [62, 63].

**Figure 3:**
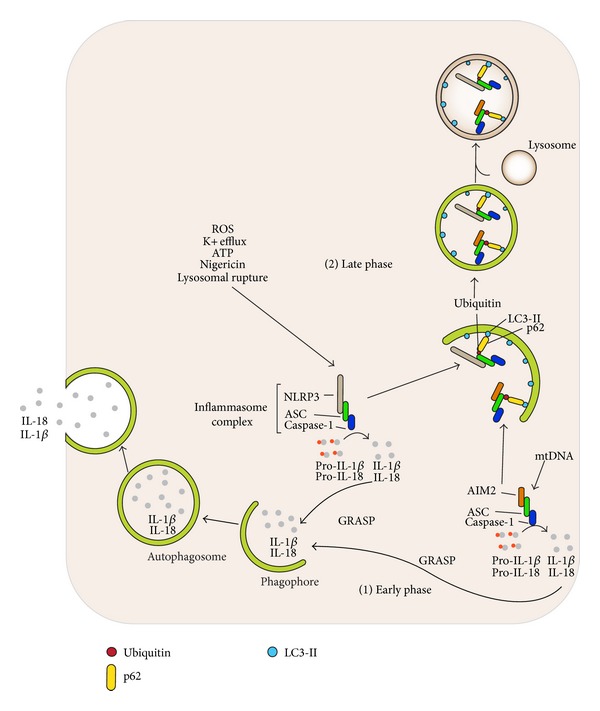
The regulation of early and late phases of inflammasome activity through the autophagic process is shown. Distinct inflammasome complexes are assembled by a variety of different stimuli. For example, reactive oxygen species (ROS), adenosine triphosphate (ATP), potassium efflux, nigericin, and lysosomal rupture trigger the activation of the sensor molecule NLRP3, whereas mitochondrial DNA (mtDNA) and pathogen-associated DNA activate the sensor molecule AIM2. The activation of sensor molecules leads to their oligomerization and further assembly of inflammasome complexes by recruiting adaptor protein ASC and procaspase-1 leading to the cleavage of the proform. Activated caspase-1 then cleaves the proinflammatory cytokine precursors prointerleukin-1*β* (pro-IL-1*β*) and pro-IL-18 into biologically active forms of IL-1*β* and IL-18. (1) At the early phase of inflammasome activation, biologically active forms of IL-1*β* and IL-18 are transported into autophagic vesicles via GRASP proteins and secreted outside of the cell through autophagic vesicles. Hence, autophagic pathway regulates inflammasome activity by contributing the secretion of IL-1*β* and IL-18. (2) In the late phase, inflammasome complexes are selectively degraded by autophagic vesicles. The multimeric inflammasome structures are ubiquitinated; one target is the adaptor protein ASC. The autophagic adaptor protein p62 mediates the recruitment of ubiquitinated inflammasomes as autophagic cargo into autophagic vesicles. Inflammasome structures are later degraded by hydrolytic enzymes following lysosomal fusion. Hence, the autophagic pathway acts to limit inflammasome activity by engulfing and degrading them.

In addition to the degradation role of autophagy, several studies have underscored its role in the unconventional secretion of leaderless proteins that cannot enter the ER and lack signal sequences required for standard secretion [10, 64]. These proteins can be secreted by an autophagy-dependent pathway [10, 65]. The extracellular secretion of pro-IL-1*β* and IL-18 during inflammasome activation is mediated by such an unconventional secretion mechanism. The robust activation of nonselective autophagy pathways by starvation at the early stages of nigericin-induced inflammasome activation elevated the amount of secreted IL-1*β* and IL-18 in an ATG5, Rab8a, and GRASP55 dependent fashion [65]. The inflammasome end products IL-1*β* and IL-18 are transported to extracellular space via autophagic vesicles formed upon starvation. ATG5 seems to be an essential protein for starvation-induced autophagy initiation, whereas Rab8a, a vesicular transport protein, and GRASP55, Golgi reassembly stacking protein, are required for efficient autophagy-dependent secretion of IL-1*β* [66]. Together these studies indicate that autophagy has a dual role in the regulation of inflammasome activity (Figure 3). Initially, autophagy governs the unconventional secretion of inflammasome products, but at later stages autophagy acts to selectively degrade inflammasomes [10].

## 3. Bacterial Infection and Autophagy (Xenophagy)

The discovery of the linkage between microbial infection and autophagic activation has led to the identification of additional autophagic adaptors and of regulatory mechanisms that specifically target, attack, and degrade various bacteria. The autophagic response against intracellular pathogens (bacteria, viruses, fungi, and parasites) is named xenophagy. Xenophagy often proceeds by the selective uptake of invading microorganisms via signals, autophagic adaptors, and receptors, which delivers the bacteria to the autophagosomes [9, 67]. Not only invading pathogens but also aggregation-prone proteins and damaged organelles are recognized and captured by specific autophagic adaptors [5]. These adaptor proteins are termed sequestosome 1/p62-like receptors (SLRs). Besides p62, other identified SLRs include NBR 1, NDP52 (nuclear dot protein 52), and optineurin proteins [18, 68]. The SLRs include an LC3 interacting region (LIR motif) and one or more cargo recognition domains that recognize ubiquitin-tagged or galectin-tagged targets. LIR domain of SLRs provides a means to link to autophagosomes, whereas the ubiquitin binding domain functions in cargo recruitment such that the SLR protein builds a bridge between the autophagosomes and modified microorganism or other targets [68]. Some SLRs have an inflammation-associated domain, which interacts with proinflammatory factors. Receiving such signals improves the SLRs ability to recognize cargo, enhances autophagy, and facilitates target degradation [9]. The number of SLRs and the types of unique structures they recognize will likely grow, as they are the continued focus of numerous investigative efforts.

The p62 protein is involved in cell signaling, receptor internalization, and protein turnover [69–72]. It specifically targets polyubiquitinated* Salmonella typhimurium* and* Shigella flexneri* to autophagosomes and restricts their intracellular growth, hence endowing antimicrobial activity to autophagosomes [73, 74].* Shigella* also recruits NEMO and TRAF6 to* Shigella* vacuolar membrane remnants, whereby p62 interacts with polyubiquitinated TRAF6 [75]. p62 and NDP52 target* Shigella* to a septin and actin dependent autophagy pathway while these same proteins target a* Listeria* mutant to a different autophagy pathway, one not dependent upon septin and actin. This indicates a degree of specialization among the selective autophagy pathways [73]. p62 also interacts with the Sindbis virus capsid protein, which targets the virus to autophagosomes during a Sindbis infection of the mouse central nervous system [76].

Another adaptor protein NDP52 recognizes the ubiquitin-coated* Salmonella enterica* and it recruits TBK-1 (tank-binding kinase) to* S. typhimurium* [77]. During a* Salmonella* infection knockdowns of either TBK-1 or NDP52 enhance bacterial growth and elevate the amount of ubiquitin-coated cytosolic* Salmonella* [78, 79]. Additionally, TBK-1 phosphorylates the SLR optineurin following its recruitment to ubiquitinated cytosolic* Salmonella*, thereby enhancing LC3 binding [80]. Knockdown of each adaptor protein enhances* Salmonella* replication as each binds a different type of ubiquitin chain and localizes to a distinct bacteria microdomain [9]. Also, p62 can be phosphorylated by TBK-1 at Ser-403, which increases the affinity of p62 for polyubiquitin chains. This has been shown to improve autophagosome maturation and the autophagy-dependent elimination of* Mycobacterium tuberculosis* var.* bovis *BCG [78, 81].

Following cytosolic invasion, many intracellular pathogens escape vacuolar membranes. This exposes previously unexposed glycans on the pathogen-damaged host membranes. When* Salmonella *escapes from vacuolar membranes, the intracellular lectin galectin-8 binds to the exposed *β*-galactoside containing glycans. This recruits the SLR NDP52 via its galectin-interacting region motif, which links the disrupted vacuolar membrane to LC3 on the isolation membrane. Galectin-8 acts as a restriction factor to limit the growth of the escaped* Salmonella* [82–84]. Furthermore, when* Salmonella* escapes from vacuolar membranes, they become targets of the E3 ligase LRSAM1, which directly ubiquitinates the bacteria. This results in the ubiquitin dependent recruitment of NDP52 and p62 to the bacteria and their delivery to autophagosomes [85].

### 3.1. Phagocytosis and Autophagy

**Figure 4:**
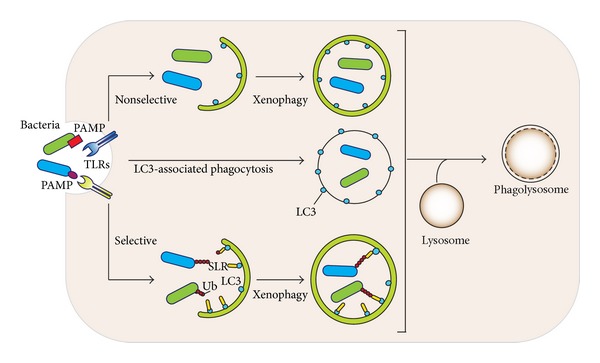
The autophagic response against intracellular pathogens (xenophagy) is shown. Xenophagy is initiated by the recognition of various PAMPs of different bacteria by corresponding TLRs. The invading microorganisms are phagocytized and delivered to autophagosomes. Xenophagy proceeds as either a nonselective or selective uptake of bacteria via signals, autophagic adaptors, and receptors. For the selective uptake, ubiquitinated bacteria are recruited into autophagosomes via sequestosome 1/p62-like receptors proteins. Another means of xenophagy is LC3-associated phagocytosis, which represents the recruitment of LC3 to phagosomes following TLR activation. LC3 recruitment to such phagosomes triggers the fusion with lysosomes. All three different xenophagy pathway ends with lysosomal fusion leading to degradation of the engulfed pathogen.

Macrophages attempt to eliminate extracellular bacteria and materials by phagocytosis, which is defined as the internalization of large particles such as cellular debris, apoptotic cells, and pathogens into phagosomes [86]. The contents of the phagosomes can be degraded by the fusion of phagosomes with late endosomes and/or lysosomes [67]. Not surprisingly autophagy and phagocytosis mechanistically overlap [87]. For example, TLR signaling enhances the maturation of phagosomes and also increases entrapment of* Mycobacterium* in autophagosomes [88]. LC3, a critical component in the autophagy pathway, can be recruited to phagosomes following the exposure of macrophages to TLR agonist-coated beads or zymosan. This process has been termed “LC3-associated phagocytosis (LAP).” LAP depends upon high levels of PI3K activity and an initial recruitment of Beclin-1 onto the phagosomes. This is followed by association of LC3 with phagosomes and further acidification. The localization of LC3-II on the phagosomal membrane has been documented by proteomic studies analyzing the composition of phagosomal membranes [89]. TLR-induced LC3 recruitment to the phagosome does not depend upon the induction of autophagy. However, ATG5 and ATG7 are required for LC3 localization on the phagosome following TLR stimulation. In contrast ULK1, a kinase required for the initiation of classical autophagy pathway, has no role in LAP. In addition, LAP helps macrophages clear apoptotic and necrotic cells, thereby eliminating potential triggers of autoimmunity [90]. A recent study revealed another interaction between the pathways leading to autophagy and phagocytosis. ATG7-deficient macrophages were found to have increased levels of class A scavenger receptors—macrophage receptor with collagenous structure (MARCO) and macrophages scavenger receptor 1 (MSR1)—because of the accumulation of p62 [91]. The upregulation of these receptors led to higher phagocytic uptake rates and increased bacterial uptake revealing that the loss of autophagy can enhance phagocytosis [92].  Figure 4 highlights the xenophagy and LAP pathways.

## 4. Concluding Remarks and Perspective

The macrophage innate immune response and autophagic processes are closely connected and modulated by TLR activation, inflammasome activation, and bacterial infection. Although much is known, further research is needed to answer a number of important questions. A few of the many questions are listed below. As autophagy is intimately involved in the innate immune response and in responding to nutritional energy status of the cell, how do these pathways interrelate? During starvation AMBRA1, a component of Beclin-1 complex, recruits TRAF6, which stabilizes the self-association of ULK1 proteins through polyubiquitination [72]. Does TRAF6 similarly affect ULK1 in TLR-activated macrophages? RalB is a small GTPase that engages two components of the exocyst complex, EXO84 and SEC5. RalB-EXO84 interactions lead to assembly of ULK1 and PI3KC3 upon initiation of autophagosome formation, whereas RalB-SEC5 induces innate immune signaling [93]. What are the upstream elements leading to RalB activation? How do signals that trigger inflammasomes also induce RalB activation and autophagy? Another question is how phagophores surround ALIS formed following LPS treatment of macrophages without a requirement for ATG5 and ATG7. While an ATG5/ATG7-independent alternative macroautophagy pathway has been discovered [43], the molecular events leading to closure of the phagophore and elimination of ALIS structures following TLR-induction remain enigmatic. Given the diversity and nonredundancy of autophagy adaptors, do adaptors other than p62 target the ubiquitinated inflammasome complexes and regulating inflammatory response? If so, then what are the spatio-temporal mechanisms that control ubiquitin-specific selective autophagy during TLR-induced, inflammasome-induced, and bacterial infection-induced autophagy? Growth factor- and G protein-mediated signaling pathways are also shown to regulate the intracellular autophagic balance in addition to the essential components of the autophagic process. According to recent findings of our group, such signaling pathways do not seem to affect macrophage autophagic activity suggesting differential tissue/cell type regulation of autophagy [94]. Related to that, one may ask are there any other specific signaling pathways regulating the autophagic balance of macrophages? Elucidating the mechanisms of autophagy/innate immunity crosstalk may facilitate the development of context-dependent therapeutics for certain inflammatory diseases and bacterial infections.
